# Treatment With LAU-7b Complements CFTR Modulator Therapy by Improving Lung Physiology and Normalizing Lipid Imbalance Associated With CF Lung Disease

**DOI:** 10.3389/fphar.2022.876842

**Published:** 2022-05-20

**Authors:** Amanda Centorame, Daciana Catalina Dumut, Mina Youssef, Martin Ondra, Irenej Kianicka, Juhi Shah, Radu Alexandru Paun, Tomas Ozdian, John W. Hanrahan, Ekaterina Gusev, Basil Petrof, Marian Hajduch, Radu Pislariu, Juan Bautista De Sanctis, Danuta Radzioch

**Affiliations:** ^1^ Faculty of Medicine, McGill University, Montreal, QC, Canada; ^2^ Infectious Diseases and Immunity in Global Health, Research Institute of the McGill University Health Centre, Montreal, QC, Canada; ^3^ Institute of Molecular and Translational Medicine, Faculty of Medicine and Dentistry, Palacky University, Olomouc, Czechia; ^4^ Czech Advanced Technology and Research Institute, Palacky University, Olomouc, Czechia; ^5^ Laurent Pharmaceuticals, Montreal, QC, Canada; ^6^ Department of Biomedical Engineering, McGill University, Montreal, QC, Canada; ^7^ Department of Physiology, McGill University, Montreal, QC, Canada; ^8^ Meakins-Christie Laboratories, The Centre for Respiratory Research at McGill University and the Research Institute of the McGill University Health Centre, Montreal, QC, Canada

**Keywords:** cystic fibrosis, LAU-7b, TRIKAFTA, ceramides, fenretinide (4-HPR), sphingolipids, triple therapy, lung physiology

## Abstract

Cystic fibrosis (CF) is the most common autosomal recessive genetic disease in Caucasians, affecting more than 100,000 individuals worldwide. It is caused by pathogenic variants in the gene encoding *CFTR*, an anion channel at the plasma membrane of epithelial and other cells. Many CF pathogenic variants disrupt the biosynthesis and trafficking of CFTR or reduce its ion channel function. The most frequent mutation, loss of a phenylalanine at position 508 (F508del), leads to misfolding, retention in the endoplasmic reticulum, and premature degradation of the protein. The therapeutics available for treating CF lung disease include antibiotics, mucolytics, bronchodilators, physiotherapy, and most recently CFTR modulators. To date, no cure for this life shortening disease has been found. Treatment with the Triple combination drug therapy, TRIKAFTA^®^, is composed of three drugs: Elexacaftor (VX-445), Tezacaftor (VX-661) and Ivacaftor (VX-770). This therapy, benefits persons with CF, improving their weight, lung function, energy levels (as defined by reduced fatigue), and overall quality of life. We examined the effect of combining LAU-7b oral treatment and Triple therapy combination on lung function in a F508del^tm1EUR^ mouse model that displays lung abnormalities relevant to human CF. We assessed lung function, lung histopathology, protein oxidation, lipid oxidation, and fatty acid and lipid profiles in F508del^tm1EUR^ mice.

## Introduction

Cystic Fibrosis (CF, OMIM #219700) is an autosomal recessive disorder that commonly affects Caucasians. It is caused by genetic mutations in a gene on human chromosome 7 which codes for an epithelial membrane protein that acts as a cAMP-activated ATP-gated chloride and bicarbonate channel and water transport regulator, known as the cystic fibrosis transmembrane conductance regulator (CFTR) (Rommens et al., 1989). Mutations affecting CFTR gene expression result in difficulties with the processing, folding, or trafficking of the protein to the membrane which adversely affect its function. Among the 2000 different genetic variants causing the disease, the most common is F508del, a deletion of the codon for phenylalanine at position 508. This mutation leads to abnormal folding of the CFTR protein and ultimately, degradation of F508del-CFTR occurs mostly at the proteasome, after ER retention, which prevents its trafficking to the Golgi (Naehrig et al., 2017). Missing CFTR chloride channels at the plasma membrane result in liquid depletion and acidification of the airway surfaces impacting mucus clearance (Ratjen et al., 2015; Veit et al., 2016).

Phenotypically, CF manifestations are extremely variable and can be observed in multiple organs such as the pancreas, the gastrointestinal tract, the reproductive system, and most prominently the lungs (Ratjen et al., 2015; Veit et al., 2016). Moreover, Weber and others demonstrated a link between defective CFTR protein and its implications in the activation of the NF- κ B pathway, associated with the drastic increase of IL-8 (Weber et al., 2001; Rottner et al., 2009).

In addition to cytokines, the impaired inflammatory response in CF reflects an imbalance between the initiation and the resolution of the inflammatory response, with the overexpression of pro-inflammatory cytokines and mediators, while anti-inflammatory and pro-resolving mediators are suppressed. During this imbalance in homeostasis, pro-inflammatory lipid mediators, including 2-series prostaglandins and 4-series leukotrienes, are formed when 20-carbon, omega-6 unsaturated fatty acid, arachidonic acid (AA), is metabolized (Ricciotti and FitzGerald, 2011; Lands, 2015). On the other hand, docosahexaenoic acid (DHA), a 22-carbon unsaturated omega-3 fatty acid, is metabolized to yield anti-inflammatory resolvins and protectins (Serhan et al., 2008), which can orchestrate the timely resolution of inflammation (Kohli and Levy, 2009). Therefore, the elevated AA levels in persons with Cystic Fibrosis (PwCF) directly contribute to an increased production of pro-inflammatory mediators, meanwhile, a decrease in DHA and EPA play important roles in reducing the production of anti-inflammatory mediators (Lachance et al., 2013; Garić et al., 2020b).

It has been well documented that sphingolipid metabolism is dysregulated in CF (Guilbault et al., 2007; Guilbault et al., 2008; Guilbault et al., 2009; Garić et al., 2020b). Ceramides are produced through various pathways and the relative composition of specific species of ceramides depends on the expression of specific combination of ceramide synthases in the organs. In the lungs of healthy mice, very long-chain ceramides (VLCCs, C24:0 CER and C26:0 CER) were shown to represent more than 70% of the total ceramide pool, whereas long-chain ceramides (LCCs, C14:0 and C16:0) constitute less than 20% of the total ceramide pool (Petrache et al., 2013). The relative ratios between VLCCs and LCCs are dramatically affected in CF disease.

In 2006, Fenretinide (FEN), a synthetic retinoid, was reported to effectively inhibit IL-8 release from CFTR-deficient lung epithelial cells (Vilela et al., 2006). Subsequently, FEN has been studied in the context of inflammation in CF disease and its effects have been attributed to its overall ability to trigger the resolution phase of inflammation, as demonstrated by the modulation of macrophage-secreted inflammatory cytokines, to the correction of omega-3/omega-6 fatty acid imbalances impacting phosphorylation of ERK1/2, to the correction of the ceramide deficiency in PwCF, and to the resolution of lung mucus plugging under infection with *P. aeruginosa* (Lachance et al., 2013; Garić et al., 2020a; Veltman et al., 2021). Furthermore, Garić et al. found a partial synergistic relationship between FEN and Zinc (Zn^2+^), whose deficiency has been reported in PwCF and is associated with severity of CF lung disease (Yadav et al., 2014; Garić et al., 2017).

In 2015, a novel oral formulation of Fenretinide, LAU-7b, was used in a Phase 1, double-blinded, randomized, (3:1, active:placebo), placebo-controlled clinical study, involving 15 adult PwCF (clinicaltrials.gov, NCT02141958). The results from this trial indicated that LAU-7b was safe and well tolerated, while also normalizing lipid imbalances and reducing oxidative stress, as measured in pre-selected plasma biomarkers. Non-clinical experiments with FEN have shown to normalize the aberrant ratio between VLCCs and LCCs as well as AA/DHA ratio *in vitro* F508del-CFTR expressing cell lines and *in vivo* (LAU-7b formulation) in mouse models of CF (Lachance et al., 2013; Garić et al., 2020a; Veltman et al., 2021). It is hypothesized that the cumulated benefits of FEN, both during and after the exacerbation episodes, will translate into clinical benefits such as preservation of pulmonary function, reduced incidence and severity of exacerbation episodes, and ultimately a better quality of life for CF patients. This hypothesis is being tested in a Phase II clinical trial with LAU-7b in adult patients with CF (clinicaltrials.gov, NCT03265288).

Several drugs that increase CFTR channel activity, known as potentiators and correctors, have been developed to improve the folding and/or trafficking of the mutated CFTR protein to the plasma membrane. In July 2012, Kalydeco^®^ (Ivacaftor), the first treatment to specifically target G551D mutations causing CF (4–6% of patients), was approved by the Food and Drug Administration (FDA) in the United States (US). In 2015, it was followed by Orkambi^®^ (Lumacaftor/Ivacaftor), a combination of CFTR modulators targeting two copies of the F508del mutation (F508del/F508del). In 2018, a more effective combination, Symdeko^®^ (Tezacaftor/Ivacaftor) addressing a larger number of CF patients (30%) was released. In October 2019, the Triple-combination therapy TRIKAFTA^®^ was approved in the US for PwCF over the age of 12 with at least one copy of F508del-CFTR and recently has been expanded to include an additional 171 CFTR mutations (Table 3: List of CFTR mutations responsive to VX-445+VX-661+VX-770 (Trikafta®) and approved by the FDA) (Lopes-Pacheco et al., 2021). TRIKAFTA^®^ is composed of three drugs: Elexacaftor (VX-445), Tezacaftor (VX-661) and Ivacaftor (VX-770) which when used *in vitro* are referred to as a Triple therapy. Elexacaftor and Tezacaftor are CFTR correctors, which work to increase the amount F508del-CFTR protein that reaches the plasma membrane by aiding its processing and trafficking, while Ivacaftor is a CFTR potentiator that works at the cell surface to increase chloride channel activity (Hoy, 2019).

In this study, we examine whether the combination of LAU-7b with TRIKAFTA^®^, could have the potential of translating into a clinical benefit to PwCF.

## Materials and Methods

### F508del^tm1EUR^ Mice

The *Cftr* ^*tm1EUR*^ mouse model (C57BL/6J), heterozygous for the F508del *CFTR* mutation, was obtained from the Erasmus Medical Center (Rotterdam, Netherlands) (Wilke et al., 2011; Fontés et al., 2015). Mice were fed with standard diet and water *ad libitum*. Mice of 8–20 months were randomly assigned to five groups: 1) Non-treated, 2) Vehicle-treated, 3) LAU-7b-treated, 4) Triple drug-treated, and 5) combined Triple drug therapy and LAU-7b therapy. The treated mice received daily doses of Vehicle, LAU-7b (25 mg/kg) representing 10 mg/kg fenretinide content, Triple drugs (32 mg/kg Ivacaftor, 21 mg/kg Tezacaftor, 42 mg/kg Elexacaftor) or a combination of LAU-7b with Triple drug therapy by daily gavage *per os* (p.o) over the course of 14 days. The daily doses of Ivacaftor, Tezacaftor and Elexacaftor used in the mouse study were derived by allometric scaling from the daily dose of each individual modulator contained in TRIKAFTA^®^ that was approved for treatment in adult CF patients. Based on the preclinical data available in the NDA/BLA Multi-disciplinary Review and Evaluation (NDA 212273) for TRIKAFTA^®^, the bioavailability of the three CFTR modulators are similar in rodents and humans. Furthermore, all the derived doses used in the mouse study were below the NOAEL (no observed adverse event level) determined in the rodent toxicity studies with a duration of 28 days up to 3 months.

All animals were harvested 24 h after the last treatment. All experimental procedures were in accordance with Facility Animal Care Committee of the McGill University Health Center, Montreal, QC, Canada.

### Lung Resistance Analysis

Airway resistance was measured using a Buxco plethysmograph system (Buxco Research System, Wilmington, NC, United States), ventilators, and nebulizers (Harvard Apparatus, Holliston, MA, United States). Mice were anesthetized using a cocktail of ketamine, acepromazine and xylazine and were connected to a ventilator through tracheotomy as previously described (Kanagaratham et al., 2014). Standard invasive lung resistance measurement was done just prior to mouse harvest. A nebulizer was used to administer a saline dose followed by ascending doses (50 mg/ml to 100 mg/ml) of methacholine (MCh, Acetyl β-methyl choline, Cat: A2251, Sigma Aldrich, Saint Louis, MO, United States). The maximal resistance at each dose of MCh was determined for each mouse.

### Lung Histology Analysis

The left lung lobe of mice in each treatment group was inflated in 10% PBS-buffered formalin and kept in the solution for 48 h. The lung section was then processed, paraffinized, sectioned at 4 µm thickness, then deparaffinized, hydrated, and stained with hematoxylin and eosin (H&E). Infiltrating cells were quantified in four airways/mice lung at ×20 magnification, that of which were averaged and then normalized with the perimeter squares of the airway basement membrane as previously described (Kanagaratham et al., 2014).

### CFBE41o- Epithelial Cells Overexpressing wt-CFTR or F508del-CFTR

The human bronchial epithelial cell line CFBE41o-homozygous for the F508del mutation was shown to retain several characteristics of human CF bronchial epithelial cells (Ehrhardt et al., 2006). Parental CFBE41o- (F508del/F508del) cells are referred to as CFBE41o-(P) and have negligible expression of CFTR. CFBE41o-(P) cells overexpressing F508del/F508del are referred to as CFBE41o-(F508del). CFBE41o-(P) cells overexpressing wt-CFTR are referred to as CFBE41o-(WT). The CFBE41o-(P) cells originally generated by Prof. Dr. D. C. Gruenert (Bruscia et al., 2002), with a stable expression of wtCFTR, CFBE41o-(WT), and the CFBE41o-(F508del) (Bebok et al., 2005) were generously provided from Dr. John W. Hanrahan (McGill University at Montreal, Quebec, Canada). Cells were grown in Eagle’s Minimum Essential Medium (Wisent Bioproducts) supplemented with 10% fetal bovine serum (Wisent Bioproducts), 5% penicillin-streptomycin (Wisent Bioproducts) in a 5% CO_2_ - 95% air incubator at 37°C.

### Treatment of CFBE41o-(P), CFBE41o-(WT) and (F508del) Cell Lines for Lipid and Protein Analysis

For lipid analysis, cell lines were seeded in 100 mm plates with 1,000,000 cells in 12 ml of media and grown overnight to 80% confluence and treated the next day. They were treated with fresh drugs every 24 h, for a total time of 72 h with 1.25 μM LAU-7b (Laurent Pharmaceuticals) and 12.5 μM zinc-sulphate (Sigma-Aldrich), as well as Triple therapy composed of 3 μM Elexacaftor (VX-445), 3 μM Tezacaftor (VX-661) and 10 nM Ivacaftor (VX-770). After completion of treatment, the cell monolayer was washed twice with warmed D-PBS (Wisent Bioproducts), gently scraped with a cell scraper (Sarstedt), and pipetted into a 1.5 ml screw cap tube filled with 1 ml of 1 mM butylated hydroxyanisole (BHA) in a chloroform/methanol solution (2:1 vol/vol) for mass spectrometry lipid analysis. For protein analysis, CFBE41o-(F508del) cell line was seeded in 60 mm plates with 500,000 cells in 6 ml of media and grown overnight to 80% confluence and treated the next day. The cells were also treated for 72 h, with the same drug concentrations as indicated above for the various treatment combinations.

### Fatty Acid Analysis

Lipid analysis was done using CFBE41o-(P), CFBE41o-(WT) and CFBE41o-(F508del) cells, 25 mg of macerated lung and liver mouse tissue as well as 50 µl of plasma from each mouse, preserved in BHA and stored at −80°C. Classical purification of lipids was done as previously described by Folch et al. (Folch et al., 1957) and phospholipids were identified by thin layer chromatography extraction.

In parallel, from the chloroform fraction of the extracted lipids, the fatty acids were methylated under standard conditions and the esters were identified by gas chromatography. An Agilent Technologies 6890 N gas chromatograph (Germany) was used, equipped with a flame ionization detector and capillary column (30 m, 0.53 mm) (Agilent Technologies 6890 N, Germany), silica was used as stationary phase. The chromatographic conditions: detector temperature 280°C; injector temperature 250°C; initial column temperature 120°C for 1 min, and programmed to escalate at a rate of 10°C per minute up to 200°C and then at 4°C per minute up to the final temperature of 220°C. Nitrogen and hydrogen were used as carrier and auxiliary gas, respectively, with a flow rate of 1.3 ml/min. To perform the determination, 1 μl of the derived sample was injected, alternatively with a sample volume/internal standard ratio of 80/20. Fatty acids were identified by comparing the retention times and relative retention times of the standards with those of the samples purchased from Sigma Aldrich. The results obtained in mg/100 g of the sample were calculated according to AOCS methodology (AOCS 2017). Output signals were monitored using Agilent Chem Station for GC systems, data analysis and A/D converter 35900E. The data were estimated by automated integration of the area under the resolved chromatographic profile.

### Ceramide Analysis

Tubes were mixed vigorously and centrifuged at 4°C for 5 min at 3,000 rpm. The organic phase was recovered and evaporated using a Speedvac. The extracted lipids were separated as previously described (Guilbault et al., 2008; Guilbault et al., 2009; Garićet al., 2017; Garić et al., 2020b). Total ceramides were measured by ELISA after TLC purification, whereas quantification of the specific ceramides’ species among purified ceramide pool were quantified using mass spectrometry. LC-MS/MS was carried out using a TripleTOF 5600+ mass spectrometer (AB sciex) coupled to a Dionex UltiMate 3000 LC-system. The separation column was Kinetex 2.1 × 50 mm C18, guarded with a SecurityGuard 4 × 2.0 mm C18 guard pre-column (Phenomenex). The mobile phases were MilliQ water with 50 mM ammonium acetate and 0.1% formic acid (A) and isopropanol/acetonitrile (4:3) with 50 mM ammonium acetate and 0.1% formic acid (B), The flow rate was 300 μl/min and the gradient was set up to 15 min run time with first 1.5 min running 15% B, then increasing to 85% B in 4.5 min, further increase to 100% B in 12 min and decrease to 15% B in 15 min. The injection volume was 10 µl using µl pick-up option and 15% B as loading buffer using 20 µl sample loop. The sampler solvent was pure isopropanol to prevent sample carry over between runs. The MS was run in positive mode using AB Sciex DuoSpray ion source. The ion source was set up to ion source gas flow 1 to 45, gas flow 2 to 40, curtain gas to 30, temperature to 200 and ion source voltage to 4,500 V. The instrument was run in product ion mode with eleven separate experiments, one per each monitored analyte. Lipid standards were purchased from Sigma Aldrich and Avanti Polar Lipids.

### Analysis of Lipid and Protein Oxidation

Lipid peroxidation was measured fluorometrically using 2-thiobarbituric acid-reactive substances (TBARs species) as the end product of lipid peroxidation (Niehaus and Samuelsson, 1968; Ohkawa et al., 1979). Briefly, the samples of cells or macerated lung tissue were mixed with 8.1% sodium dodecyl sulfate, 20% acetic acid, and 0.8% 2-thiobarbituric acid. After vortexing, the samples were incubated for 1 h at 95°C after which butanol-pyridine was added at a 15∶1 (*v/v*) ratio. The mixture was shaken for 10 min and then centrifuged. The butanol-pyridine layer was measured fluorometrically at 552 nm after excitation at 515 nm (OptiPlate Perkin-Elmer United States). The results are expressed in nmoles of malondialdehyde (MD) (TBARs species) per mg of protein in the samples reflecting all thiobarbituric acid reactive substances (Lykkesfeldt, 2007). Oxidative damage of proteins was assessed using 3-nitrotyrosine as a surrogate marker. 3-nitrotyrosine (3-NT) was determined by ELISA as previously described using well-characterized antibodies (Ye et al., 1996; Montes de Oca et al., 2008). The antibodies (mouse IgG monoclonal, polyclonal against 3-nitrotyrosine and polyclonal goat anti-rabbit IgG-peroxidase) were obtained from Upstate Biotechnology (Lake Placid, NY). The quantification of 3-NT was performed using a standard curve with known concentrations of 3-NT from chemically modified bovine serum albumin. The sensitivity of the assay was 50 pg/ml.

### Western Blot Analysis of CFBE41o-(F508del)

CFBE41o-(F508del) cells were lysed in homemade RIPA buffer (50 mM Tris at pH = 7.4, 150 mM NaCl, 50 mM NaF, 0.2 mM Na_3_VO_4_, 0.1% SDS, 2 mM EDTA, 1% Triton-X, 0.5% Na-deoxycholate) with freshly dissolved protease inhibitor tablet (Sigma # 4693132001). The protein concentration was determined using BCA Protein Assay Kit (Thermo Scientific #23227) and proteins were denatured using 4x Laemmli Sample Buffer (Bio-Rad, #1610747) with 10% (v/v) *2-*mercaptoethanol (1x final) and RIPA. 20 µg of total protein was loaded on a gel and proteins were separated on a precast 4–15% polyacrylamide gradient gel (Bio-Rad, #4561085) by SDS-PAGE. Proteins were transferred onto polyvinylidene difluoride (PVDF) membrane using semi-dry transfer and the fast semi-dry transfer buffer (1X, final) containing 48 mM Tris, 15 mM HEPPS with freshly added sodium bisulfate (1 mM final), EDTA (1.0 mM final) and 4 N, N-dimethylformamide (1.3 mM final) as previously described (Garić et al., 2013). Membranes were incubated with primary antibodies: against the R domain of CFTR (23C5, provided by Dr. John Hanrahan lab) and against β-Actin (Santa Cruz, #sc-1616). Membranes were then incubated with secondary antibodies: goat anti-mouse (IgG-HRP, sc-2005). Lastly, membranes were developed using chemiluminescent kit (Bio-Rad, #170-5060).

### Statistical Analyses

All statistical analyses were performed using GraphPad Prism 9 (GraphPad, San Diego, CA, United States). For lung function, a two-way ANOVA with Sidak. For infiltrating cells in the airways of the mice, a two-way ANOVA with Bonferroni correction was performed. As for the analysis of the oxidation markers, fatty acids, VLCCs and LCCs, a Brown-Forsythe and Welch one-way ANOVA with Dunnett T3 correction was used. For the analysis of the VLCC/LCC ratio, a Kruskal–Wallis one-way ANOVA with Dunn’s correction was performed. Lastly, for Western Blot quantification, a two-way ANOVA with Sidak correction was done.

## Results

### Combinatory Treatment of Triple Therapy and LAU-7b Normalizes Lung Function in Homozygous F508del/F508del Mice Comparably to WT Controls

**FIGURE 1:**
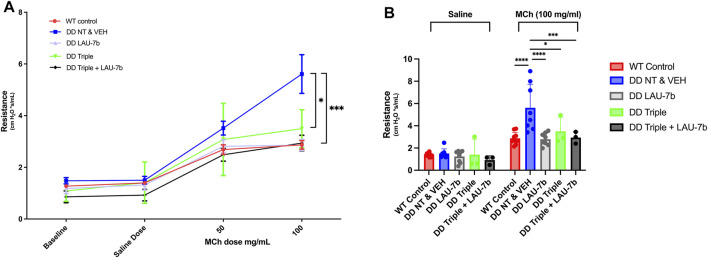
Airway function is significantly improved in F508del/F508del (DD) mice upon treatment with Triple therapy, and further improved by addition of LAU-7b, in response to MCh challenge. Treatment groups include Wild-type (WT) control (Non-treated & Vehicle, NT and VEH) (Red), DD NT and VEH (Blue), DD LAU-7b (Grey), DD Triple therapy (Green) and DD Triple + LAU-7b (Black). **(A)** Comparisons shown between DD NT and VEH and DD Triple (*p* = 0.0118) and between DD NT and VEH and DD Triple + LAU-7b (*p =* 0.0003). **(B)** Detailed analysis of the saline and 100 mg/ml MCh dose among the treatment groups. *n* = 3–12 mice for each group. Two-way ANOVA with Sidak correction where *p < 0.05, **p < 0.005, ***p < 0.0005 and ****p < 0.0001.

Lung function of F508del/F508del (DD) mice and WT mice treated with LAU-7b, Triple therapy, the combination of the two, or vehicle, was assessed using a classical lung resistance evaluation following aerosolised challenge of the lung with increasing concentrations of methacholine. Lung resistance of 14–20-month-old DD mice compared to their age-matched WT controls (red) is shown in Figure 1A. While treatment with Triple therapy (green) decreases the airway resistance in DD mice, treatment with LAU-7b (grey) shows lower airway hyperresponsiveness than all other treatment groups, comparable to WT control mice. Triple therapy alone, LAU-7b alone and their combination (black) shows protective effects at the level of lung function. Lung function was improved to a higher extent by combinatory treatment with Triple and LAU-7b than Triple therapy alone, as demonstrated by a lower airway resistance in this group of mice compared to Triple alone. In Figure 1B, resistance values recorded for the saline and 100 mg/ml MCh doses are shown to better illustrate statistical significance observed between the treatment groups. While DD NT and VEH mice compared to Triple have a *p*-value of less than 0.05, when compared to Triple + LAU-7b combination, the *p*-value is less than 0.0005. The complete results of statistical analysis are provided in Supplementary Table 1.

**FIGURE 2:**
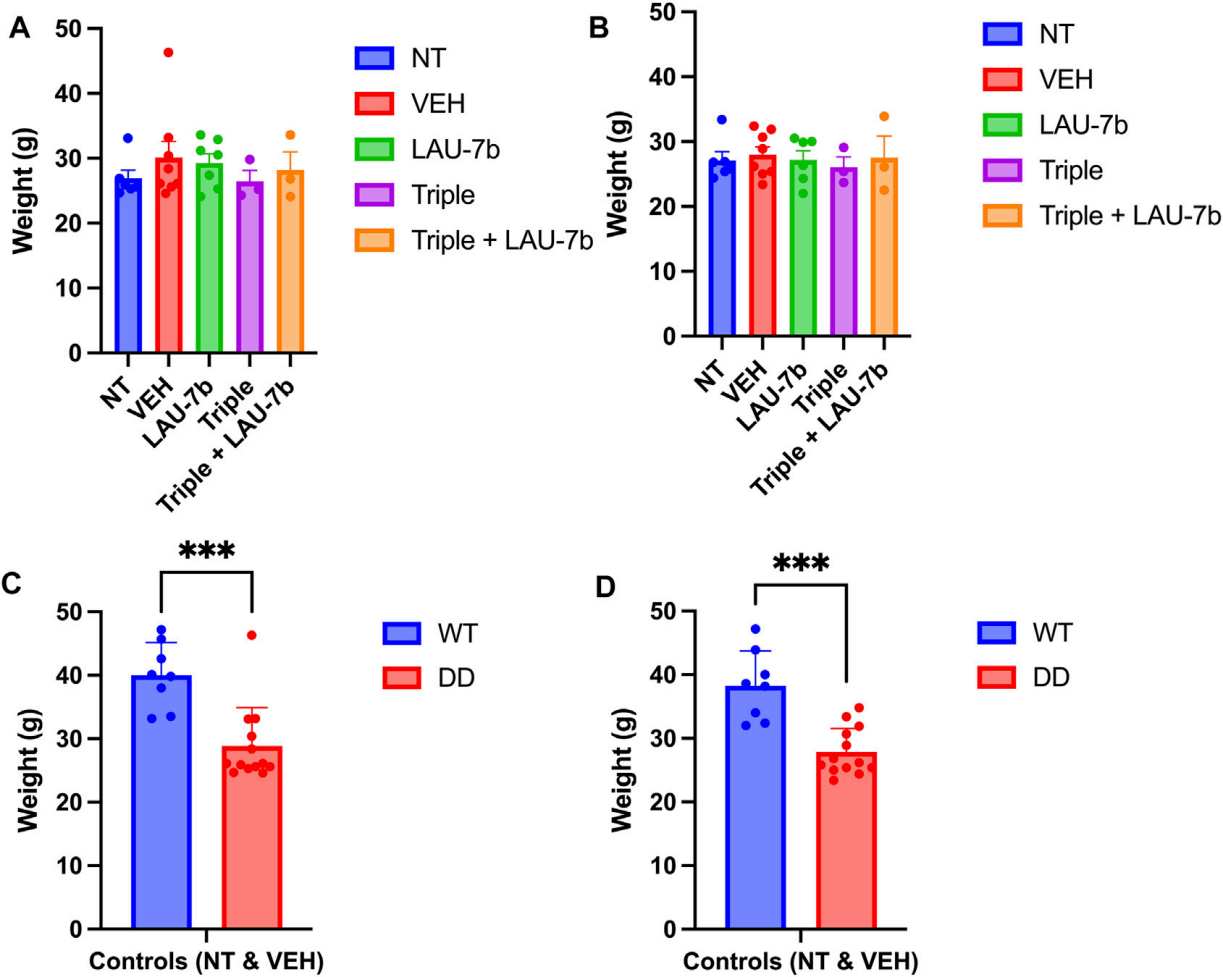
Mice weights prior to (Day 0) and following 14-day treatment (Day 15) in Wild-type (WT) and F508del/F508del (DD) mice. **(A,B)** DD mice weights (g) before treatment (Day 0) and 24 h following final treatment (Day 15) among treatment groups. There was no statistical significance between any of these groups. *n* = 3–12 mice for each group. **(C,D)** Comparison between WT and DD mice controls which include non-treated (NT), or vehicle treated (VEH) mice. *n* = 8–13 mice for each group. Welch’s *t* test was performed where *p* = 0.0003.

As shown in Figures 2A,B, the comparative analysis of weights for DD mice across all five groups does not show any statistical significance prior to the experiment (Day 0) and 24-h after final gavage (Day 15), respectively. In fact, the DD mice did not display any statistical differences in weights between treatment groups over the entire course of the oral gavage (Supplementary Figure 1). When comparing controls (non-treated, NT and vehicle treated, VEH) WT and DD mice (Figures 2C,D), as expected there was a statistically significant difference between the weights of WT and DD mice, but there was no statistically significant weight difference among the experimental groups within the same genotype.

### LAU-7b and Triple Therapy Combination Reduces Airway Hyperplasia and Immune Cell Infiltration of the Airway in the Lungs of DD Mice

**FIGURE 3:**
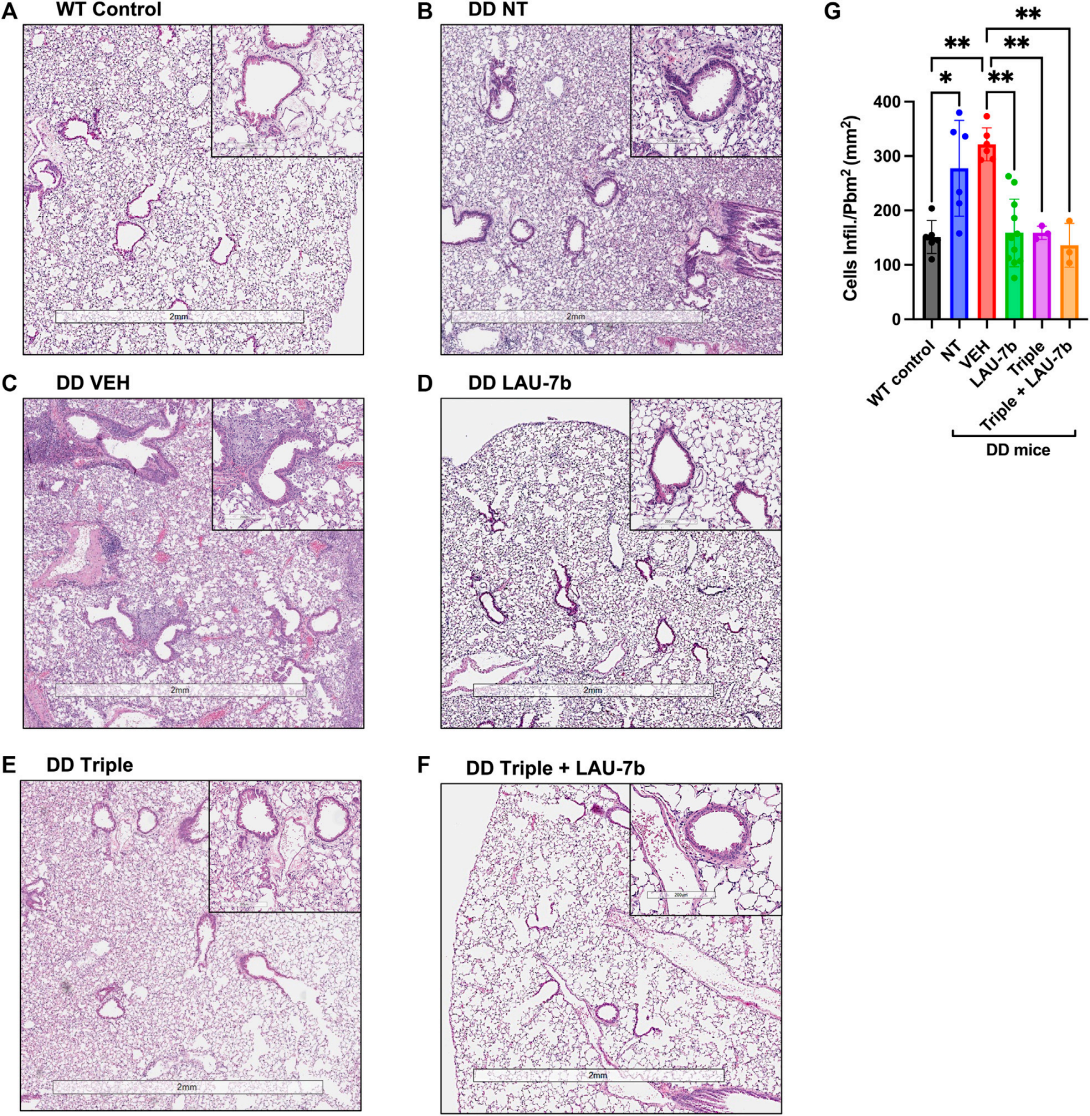
Airway hyperplasia and cellular infiltration in the lungs of Wild-type (WT) and F508del/F508del (DD) mice; Hematoxylin and eosin (H&E) staining. **(A)** WT control mice (non-treated, NT and vehicle, VEH) **(B–F)** DD NT, VEH, LAU-7b, Triple and Triple + LAU-7b, respectively. **(G)** LAU-7b and Triple therapy treated DD mice have significantly lower lung cell infiltration compared to placebo treated DD mice. For each mouse, measurements were done with at least four different airways per lung. Quantification was done by counting the number of infiltrating cells around each of the four airways per lung and normalized by dividing the square of the perimeter “in millimeter” of the airway basement membrane. *n* equal 3–11 mice for each group, Two-way ANOVA with Bonferroni correction where *p < 0.05, **p < 0.01.

After observing an improvement in airway hyperresponsiveness following combinatory treatment, we assessed lung histopathology using H&E staining (Figure 3A). DD mice were split into five treatment groups: non-treated, vehicle treated, LAU-7b, Triple therapy and LAU-7b combined with Triple therapy (Figures 3B–F). DD non-treated and vehicle treated mice with deteriorating lung function show significantly higher airway hyperplasia (Figures 3B,C). Triple therapy, as well as LAU-7b treatments alone, reduced elevated airway hyperplasia in DD mice (Figures 3D,E). The combination of the two treatments, given daily in a 14-day therapeutic cycle, resulted in further improvement and homogenous looking lungs which show no regions of hyperplasia in any of the multiple lung sections analyzed (Figure 3F). However, although a significant improvement in airway resistance following Triple therapy treatment was found, some lung sections still contain pathologically unchanged lung tissue (Figure 3E). DD mice develop thickening of the airway due to airway hyperplasia at 8-months old, without further worsening in mice 14–20 months old. As for the WT NT males, there is minimal hyperplasia seen in the airways upon comparison between 9-month-old and 14-month-old WT mice (Supplementary Figure 2). We also investigated the impacts of sex and age using our DD and WT NT mice (Supplementary Figure 2). WT and DD mice between the ages of 8–9 months and 14–20 months, both male and female, were analyzed by H&E staining of the lungs (Supplementary Figure 2). There was no difference in hyperplasia observed when comparing males and females within each experimental group. Furthermore, based on the histological assessment of Figures 3A–F, the treatments have beneficial effects on cellular infiltration in the lung parenchyma in DD mice treated with LAU-7b (Figure 3D), Triple (Figure 3E) and Triple + LAU-7b (Figure 3F) compared to DD NT and VEH treated mice (Figures 3B,C). In fact, DD mice treated with Triple and LAU-7b had lungs that look very similar to WT control mice, with the exception of hyperplasia that was still observed in some airways examined.

In addition, we evaluated airway thickening caused by cell infiltration through quantification of these cells from H&E-stained lungs (Figure 3G). A significant increase is observed between the WT control group (NT and VEH, mean value 151.08, *n* = 6, SD 30.31) and both the DD non-treated (mean value 277.5, *n* = 6, SD 87.98) and vehicle (mean value 321.6, *n* = 6, SD 30.37) treated groups. A significant decrease can be seen between vehicle treated mice compared to LAU-7b treated DD mice (mean value 158.7, *n* = 11, SD 62.18). A greater improvement was observed between vehicle treated mice (mean value 321.6, *n* = 6, SD 30.37) and Triple therapy (mean value 158.9, *n* = 3, SD 12.21), as well as with their combination (mean value 136, *n* = 3, SD 40.06). The results indicate a potential benefit with combinatory treatment of LAU-7b and Triple therapy, reducing cellular infiltration in the airways of DD mice.

### Correction of Oxidation Markers, Fatty Acids and VLCCs/LCCs in the Lungs, Liver, and Plasma of DD mice Treated with Triple Therapy and LAU-7b

**FIGURE 4:**
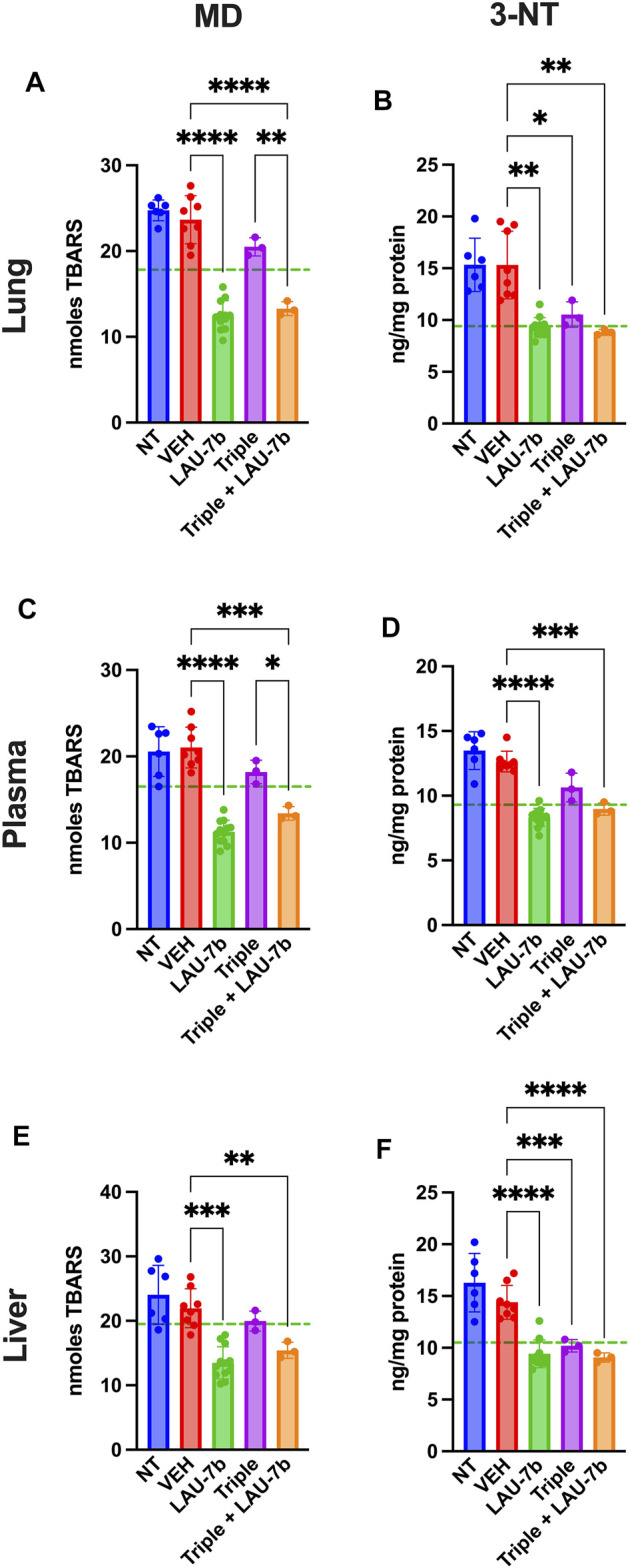
Combination of LAU-7b with Triple therapy restores the imbalances of oxidative stress markers in the lungs, plasma, and liver of F508del/F508del (DD) mice. ELISA analysis of mouse lungs **(A,B)**, plasma **(C,D)** and liver **(E,F)**. Levels for both 3-nitrotyrosine (3-NT) and malondialdehyde (MD) are significantly elevated in Non-treated (NT, *n* = 6) and vehicle treated (VEH, *n* = 8) DD mice as compared to mean levels seen in WT mice (dotted green line). LAU-7b treatment (*n* = 12) significantly decreases levels of MD and 3-NT in all organs and plasma. Triple therapy (*n* = 3) treatment on its own has minimal effect. However, combinatory treatment with LAU-7b and Triple therapy (*n* = 3) restores the decrease in 3-NT and MD seen in the LAU-7b treated mice, when compared to VEH treated DD mice. Brown-Forsythe and Welch One-Way ANOVA with Dunnett T3 correction, where *p < 0.05, **p < 0.01, ***p < 0.001, ****p < 0.0001.

Non-treated and vehicle treated DD mice show increased baseline levels of Malondialdehyde (MD), marker of lipid oxidation, and 3-nitrotyrosine (3-NT), marker of protein oxidation, compared to WT control mice (dotted green line) in lungs, liver, and plasma (Figure 4). Triple therapy, and LAU-7b treatment significantly decrease levels of MD and 3-NT, in lungs, liver, and plasma when compared to vehicle treated DD mice. Across all samples shown in Figures 4A–F, combinatory treatment of Triple with LAU-7b significantly decrease both MD and 3-NT to comparable levels with WT mice (dotted green line) or improves these levels below WT, accentuating the benefit of the combinatorial treatment in slowing CF disease progression.

**FIGURE 5:**
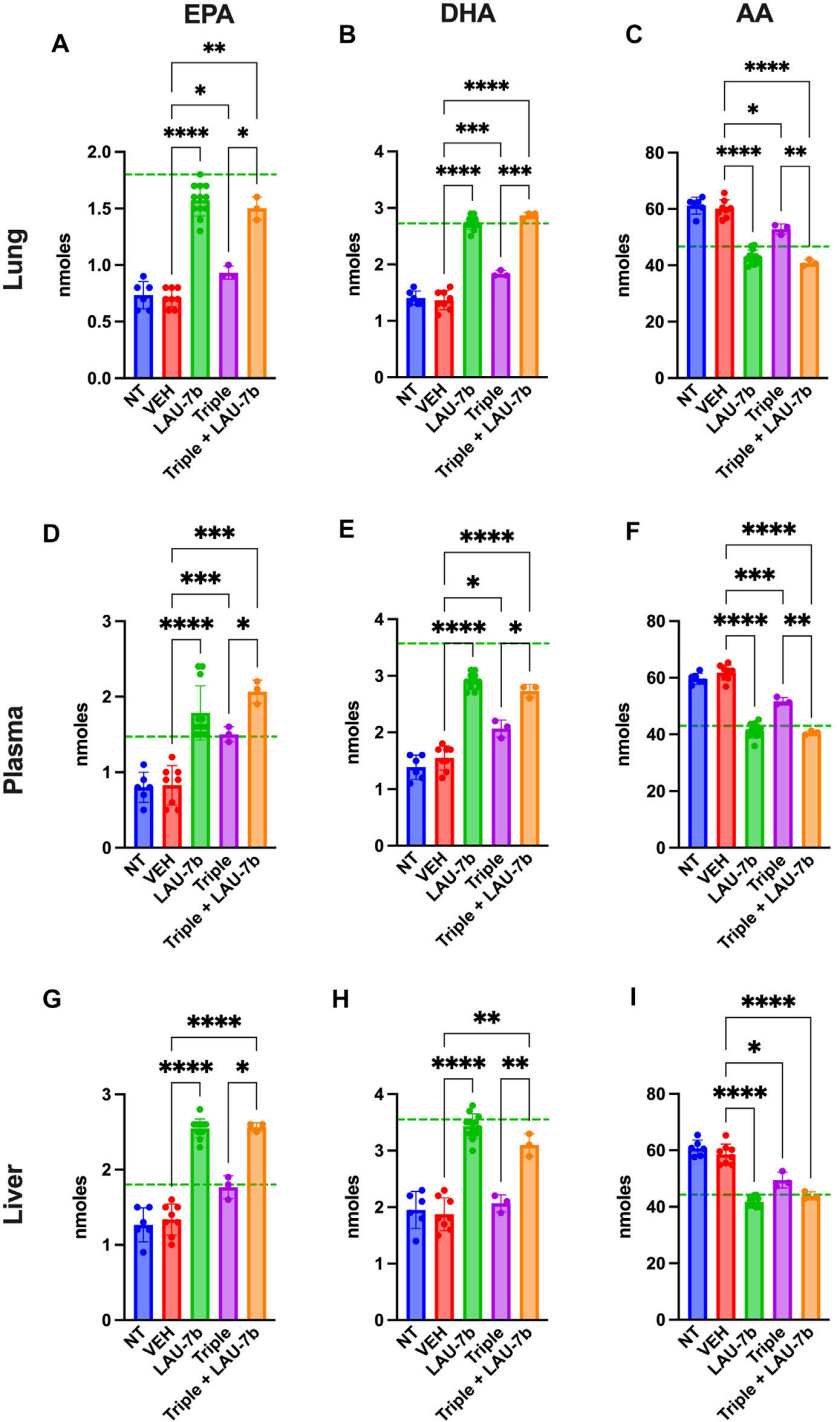
Correction of fatty acids in the lungs, plasma and liver of F508del/F508del (DD) mice is enhanced upon combination of Triple therapy and LAU-7b. Gas chromatography analysis of mouse lungs **(A–C)**, plasma **(D–F)** and liver **(G–I)** fatty acids. LAU-7b significantly increases omega-3 fatty acids, eicosapentaenoic acid (EPA) and docosahexaenoic acid (DHA) in all three samples comparably to mean levels seen in WT mice (dotted green line). While Triple therapy alone only shows limited level of correction in lungs and plasma, the combination of the two significantly increases EPA and DHA in all samples when compared to vehicle (VEH) treated DD mice. Meanwhile, omega-6 fatty acid, arachidonic acid (AA) levels are significantly decreased in both LAU-7b and Triple therapy treated DD mice seen in all samples. Further improvement is seen upon combination of the two treatments (*n* = 6, 8, 12, 3, 3, respectively for DD-NT, DD-VEH, DD-LAU-7b, DD-Triple, DD-Triple + LAU-7b). Brown-Forsythe and Welch One-Way ANOVA with Dunnett T3 correction, where *p < 0.05, **p < 0.01, ***p < 0.001, ****p < 0.0001.

In Figure 5 levels of omega-3 (AA) and omega-6 (EPA and DHA) fatty acids were investigated in DD mice lungs, liver, and plasma. EPA and DHA levels are significantly increased with LAU-7b treatment p.o. when compared to placebo treated DD mice. Combinatory treatment of LAU-7b with Triple therapy also significantly increases EPA and DHA comparably, if not higher, than what is seen in WT controls. On the other hand, AA levels are significantly decreased by LAU-7b treatment, and this decrease is further enhanced with Triple and LAU-7b combination. Overall, combination treatment drastically improves the imbalance observed in AA/DHA and AA/EPA ratios in different organs and plasma of DD mice.

**FIGURE 6:**
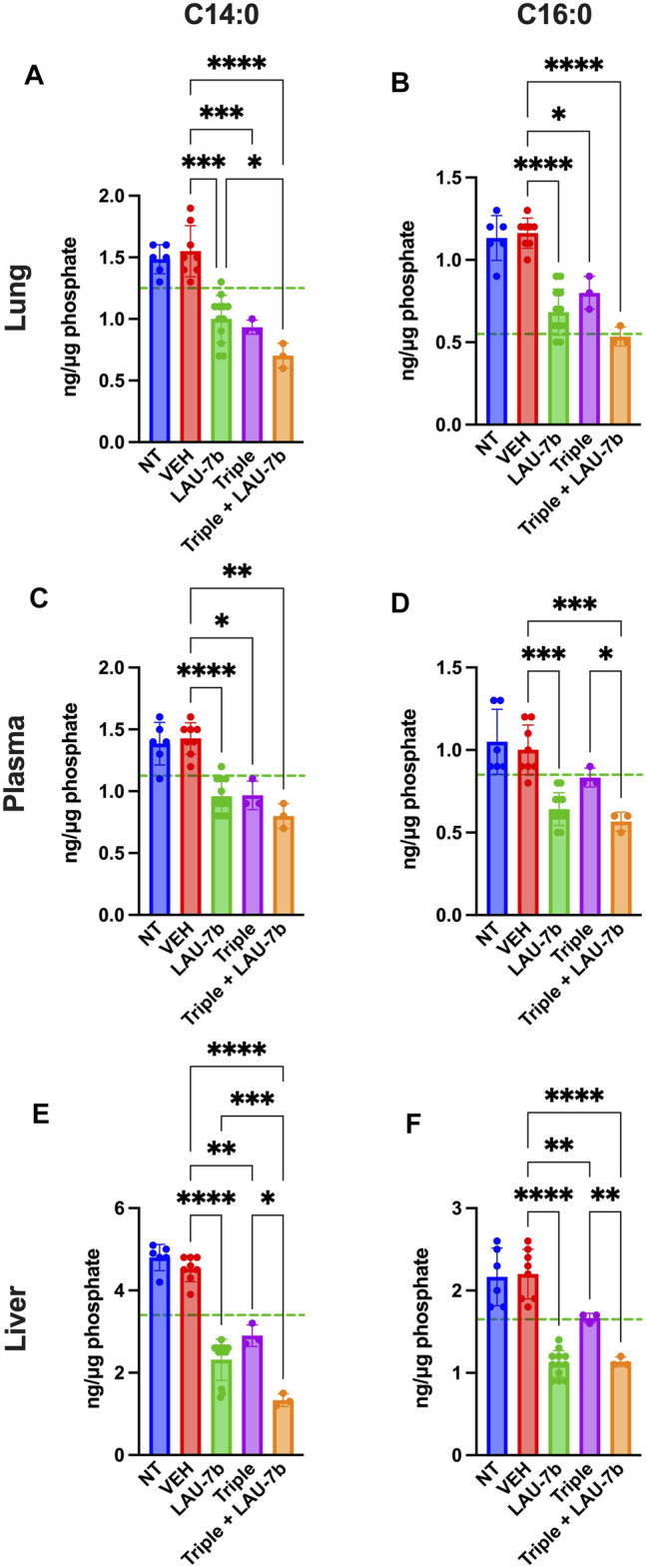
Decrease of long-chain ceramides (LCCs) is enhanced upon Triple therapy and LAU-7b combination in the lungs, plasma, and liver of F508del/F508del (DD) mice. Mass spectrometry analysis of mouse lungs **(A,B)**, plasma **(C,D)**, and liver **(E,F)**. LAU-7b treatment significantly decreases LCCs C14:0 and C16:0 when compared to vehicle (VEH) treated DD mice in all three samples below mean levels seen in WT mice (dotted green line). Similar decrease is also seen upon treatment with Triple therapy. The combination of the two treatments yields a stronger decrease in LCCs than with Triple alone in all samples (*n* = 6, 8, 12, 3, 3, respectively for DD-NT, DD-VEH, DD-LAU-7b, DD-Triple, DD-Triple + LAU-7b). Brown-Forsythe and Welch One-Way ANOVA with Dunnett T3 correction, where *p < 0.05, **p < 0.01, ***p < 0.001, ****p < 0.0001.

**FIGURE 7:**
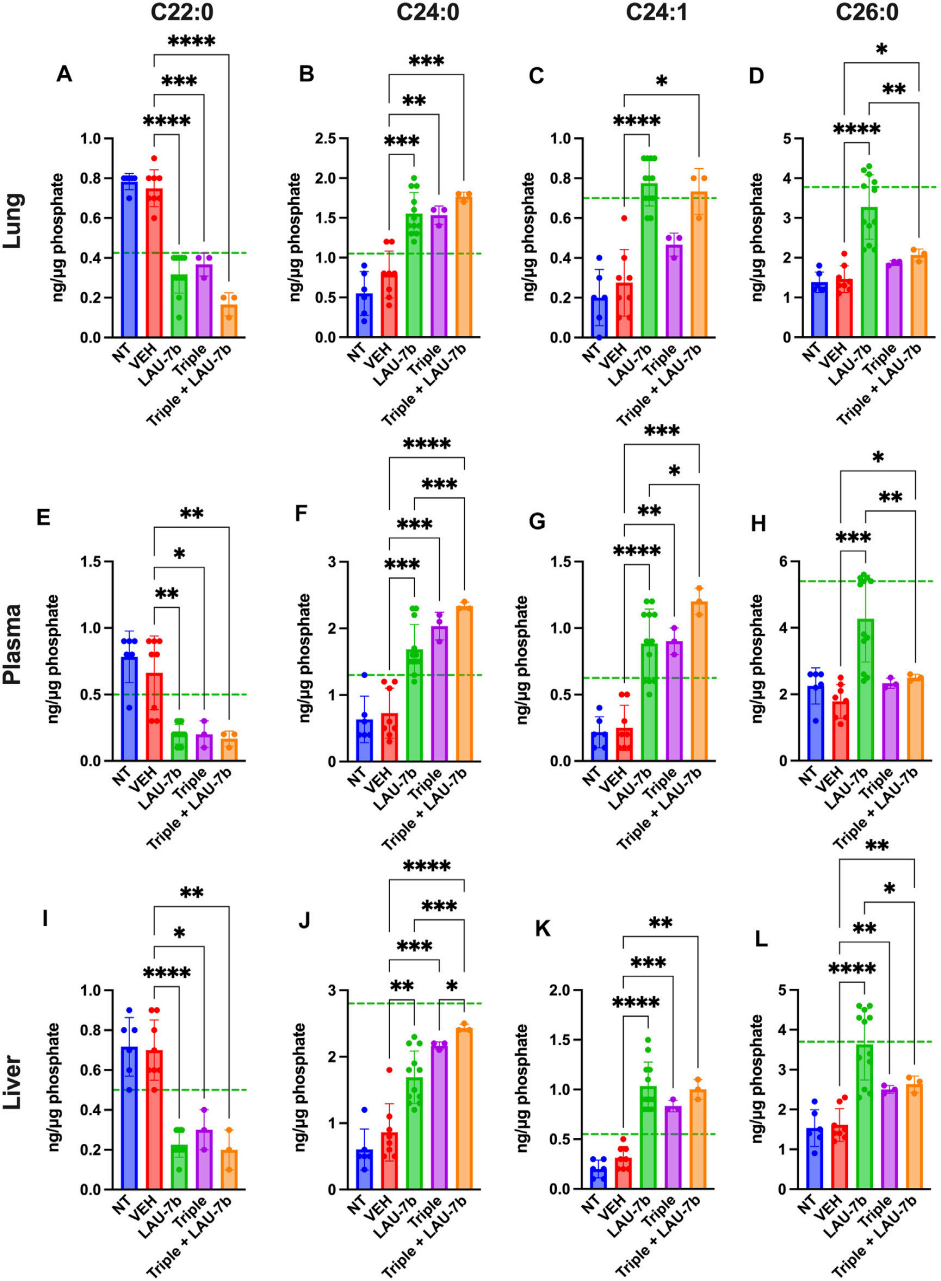
Combination of Triple therapy and LAU-7b exhibits stronger effects in correcting levels of very long-chain ceramides (VLCCs) than Triple therapy treatment alone in the lungs, plasma, and liver of F508del/F508del (DD) mice. Mass spectrometry analysis of mouse lungs **(A–D)**, plasma **(E–H)** and liver **(I–L)**. Upon treatment of LAU-7b and Triple therapy on their own, VLCC C22:0 shows significant decrease while C24:0 and C24:1 show a significant increase when compared to vehicle treated DD mice in all samples. Moreover, levels are corrected towards mean levels seen in WT mice (dotted green line). Meanwhile, only LAU-7b significantly increase C26:0 levels. Combination treatment of LAU-7b and Triple therapy show stronger effects for VLCCs C22:0, C24:0 and C24:1 than with Triple alone. However, VLCC C26:0 is somewhat increased by Triple and combination therapy but is not as strong as with LAU-7b alone (*n* = 6, 8, 12, 3, 3, respectively, for DD-NT, DD-VEH, DD-LAU-7b, DD-Triple, DD-Triple + LAU-7b). Brown-Forsythe and Welch One-Way ANOVA with Dunnett T3 correction, where *p < 0.05, **p < 0.01, ***p < 0.001, ****p < 0.0001.

Next, long-chain ceramides (LCCs), C14:0 and C16:0, which are typically elevated in DD mice as compared to WT controls (dotted green line), show improvements upon treatment with Triple therapy, or LAU-7b, both of which bring these levels below those of vehicle treated DD mice (Figure 6). Interestingly, the combination of Triple therapy with LAU-7b further decreases LCC levels below the threshold seen in WT mice across all samples. As for the very long-chain ceramides (VLCCs), Figure 7 shows a baseline increase in C22:0 and a decrease in C24:0, C24:1 and C26:0 levels in both DD non-treated mice and vehicle treated mice. These levels are normalized to the levels seen in WT mice (dotted green line) upon treatment with LAU-7b, and Triple therapy. Again, combinatory treatment significantly increases in C24:0 and C24:1 compared to WT mice while also improving C26:0 levels. Combination treatment also significantly decreases levels of C22:0, below WT levels.

**FIGURE 8:**
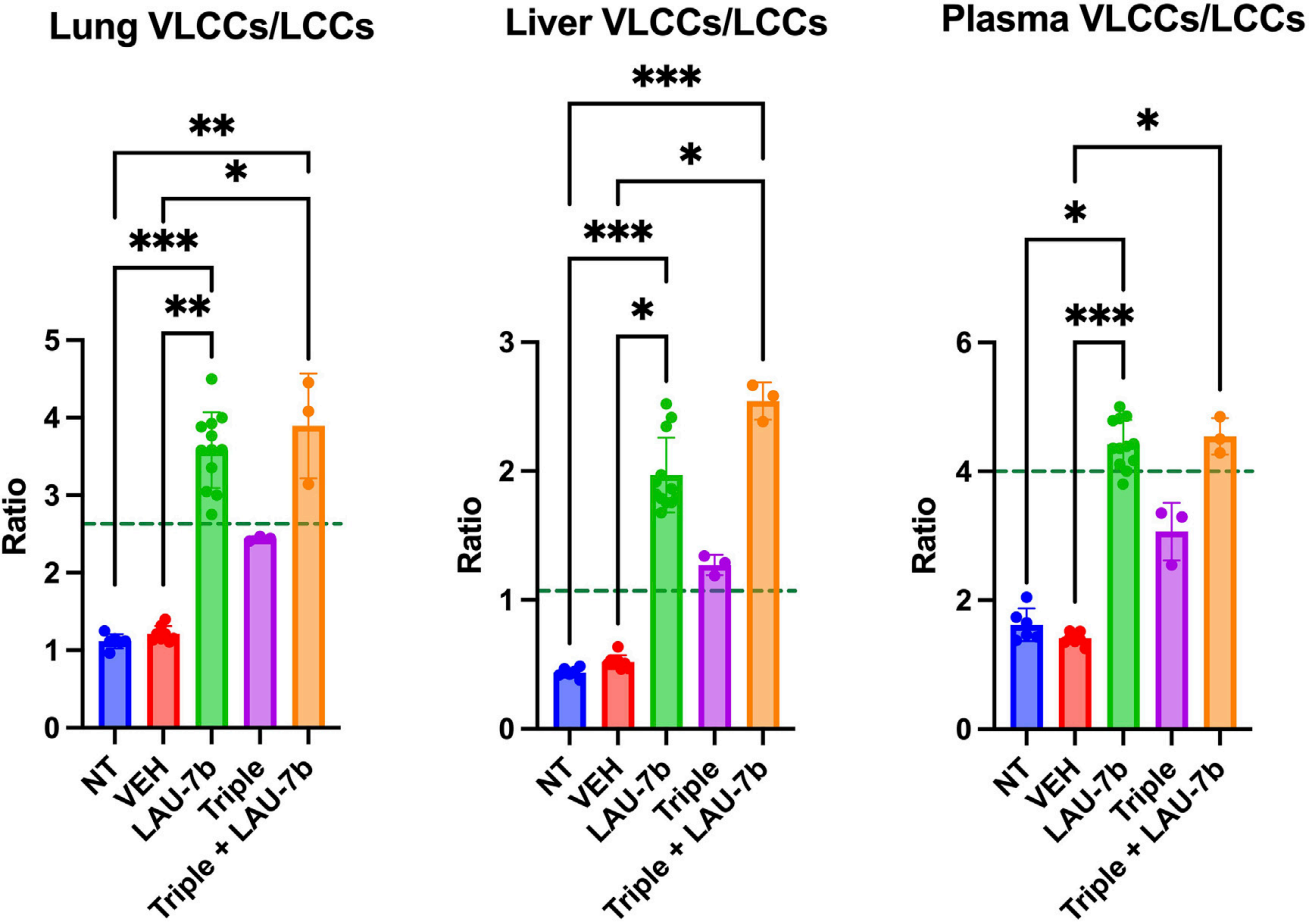
Combination of Triple therapy and LAU-7b improves VLCCs/LCCs ratios in lungs, plasma and liver of F508del/F508del (DD) mice. Treatment of LAU-7b improves the ratio of VLCC/LCC in all samples of DD mice which is higher than mean levels seen in WT mice (dotted green line). Meanwhile, the same effect is not seen in DD mice treated with Triple therapy alone. However, the combination of LAU-7b and Triple can restore and bring the VLCC/LCC ratio in lungs, plasma and liver of DD mice above the levels seen in WT mice (*n* = 6, 8, 12, 3, 3, respectively for DD-NT, DD-VEH, DD-LAU-7b, DD-Triple, DD-Triple + LAU-7b). Kruskal–Wallis One-Way ANOVA with Dunn’s correction, where *p < 0.05, **p < 0.01, ***p < 0.001, ****p < 0.0001.

Lastly, the ratios of VLCCs/LLCs for the lungs, plasma and liver of DD mice were studied (Figure 8). Treatment with LAU-7b (green) drastically improves the ratio of VLCCs/LCCs in lungs, plasma and liver of DD mice. While Triple therapy treatment may not show a statistically significant improvement, it does show a trend of correction of the ratio (purple). Moreover, the combination of LAU-7b and Triple (orange) can restore and bring the VLCC/LCC ratio above the levels seen in WT mice (dotted green line). Such correction is like that of LAU-7b′s effect on its own.

### Triple Therapy, Fenretinide and Physiological Concentration of Zinc Combination Shows Improvement in VLCC/LCC Ratio in CFBE Cell Lines

To assess causality of the combination treatment we also studied VLCC/LCC ratio improvements *in vitro*, in CFBE41o-(P), CFBE41o-(WT) and CFBE41o-(F508del) cell lines. Levels of LCCs (C14:0, C16:0), as well as VLCCs (C22:0, C24:0, C24:1, C26:0) were measured, and their ratios were obtained (Supplementary Figure 3). Similar trends are observed for CFBE41o-(P) and CFBE41o-(F508del) cell lines when it comes to their VLCC/LCC ratios. At baseline, CFBE41o-(P) and CFBE41o-(F508del) display an 8% and 19% decrease in the VLCC/LCC ratio, when compared to CFBE41o-(WT) cells (dotted line). Fenretinide and physiological levels of zinc improve VLCC/LCC ratio by approximately 2-fold when compared to vehicle, in both cell lines. Triple therapy treatment does not show improvements in VLCC/LCC ratio. However, Triple therapy in combination with fenretinide and physiological concentration of zinc restores the VLCC/LCC ratio to a similar fold increase as seen with fenretinide and zinc treatment alone, with both treatments elevating the ratio above the baseline seen in CFBE41o-(WT).

### Improvement in Total Levels a Glycosylation Status of CFTR Upon Combinatorial Treatment with FEN, Triple Therapy and Zn^2+^ in CFBE41o-(F508del)

**FIGURE 9:**
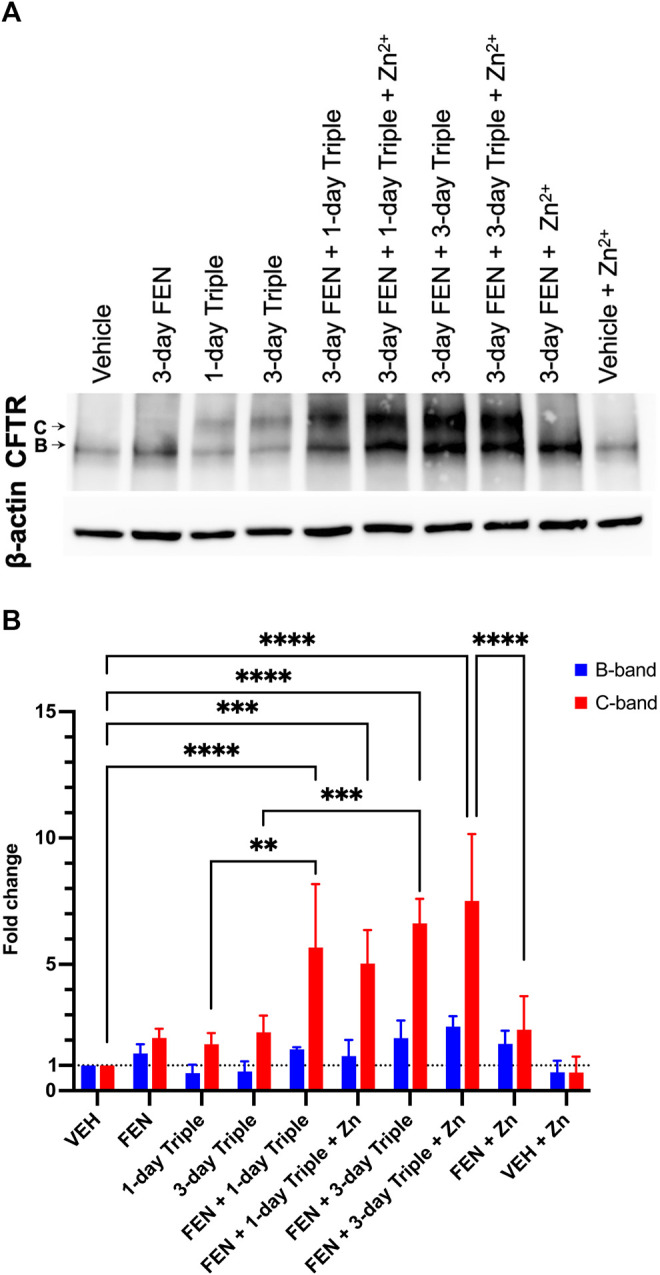
Total CFTR protein level in CFBE41o-(F508del) cell line. Western blot analysis **(A)** and quantification of three replicates **(B)**. The CFTR protein has two glycosylated forms, the B band which is ∼131 kDa, or the C band at ∼160 kDa. For both B and C bands, the greatest fold change can be seen upon combinatory treatment of FEN, Triple therapy, and Zinc. Two-way ANOVA with Sidak correction, where ***p < 0.001, ****p < 0.0001.

Given that CFBE41o-(P) does not have any detectable CFTR protein, the total levels of CFTR protein were assessed in CFBE41o-(F508del) cells treated with Triple therapy, fenretinide or the combination of both treatments, by Western blotting (Figure 9A). The B band for CFTR is ∼131 kDa, which represents the core glycosylated CFTR processed in the endoplasmic reticulum (Cheng et al., 1990; O’Riordan et al., 2000; Mall et al., 2004). The C band for CFTR, which is ∼160 kDa, represents the fully glycosylated mature CFTR and would indicate processing of the protein in the Golgi apparatus (Cheng et al., 1990; O’Riordan et al., 2000). As expected, the CFBE41o-(F508del) cell line does not express the fully glycosylated and mature CFTR. Only the B band can be observed on the immunoblot. Figure 9B illustrates the quantification data normalized to actin expression levels and shows the differences that were statistically significant. Following 3-day treatment with FEN, the B band was enhanced by 1.5-fold (50%), and upon combination of FEN with Zn^2+^, the B band is further enhanced to 1.85-fold. This indicates an increased amount of core glycosylated CFTR protein in the cells, while the fully glycosylated mature form represented by the C band remains almost undetectable. While treatment of 1-day or 3-day Triple therapy does not enhance the B band for CFTR, a substantial improvement can be seen upon 3-day treatment with FEN and Triple therapy by 2.1-fold, and upon addition of Zn^2+^, is further ameliorated to 2.6-fold increase. As for the C band, enhancement is seen for 3-day FEN treatment, Triple therapy treatment for 1 and 3-days, as well as the addition of Zn^2+^. Of all the treatments, combinatorial treatment with 3-day FEN and 3-day Triple therapy in the presence of physiological concentration of Zn^2+^, yields the highest intensity for the C band, with a 7.5-fold increase, compared to vehicle. These results demonstrate that treatment with combination of both FEN and Triple augments the total CFTR protein levels further than with Triple therapy alone. These effects can be attributed to increased protein synthesis, stabilization, and/or decreased degradation, ultimately enhancing the efficacy of the modulators. The mechanism of additive effect of this combinatory treatment should be further investigated.

## Discussion

CF impacts more than 100,000 individuals worldwide (Shteinberg et al., 2021). To date, there is no cure for CF and available treatments target its symptoms. However, CFTR modulator and potentiator therapies which target the genetic defect of the disease, have brought much hope to the CF community. Recently, two randomized, double-blind, phase 3 clinical trials involving TRIKAFTA®, for CF patients 12 years and older, heterozygous or homozygous for the F508del mutation (NCT03525444 and NCT03525548) (Heijerman et al., 2019; Middleton et al., 2019; Zah et al., 2021) were successfully completed and led to the approval of TRIKAFTA® treatment in 2019. More recently, TRIKAFTA® was approved in younger patients, 6–11 years of age, heterozygous or homozygous for the F508del mutation, following a confirmatory Phase 3 trial in this CF population (NCT03691779) (Zemanick et al., 2021). Across all three studies, improvement in percentage of predicted FEV_1_ (ppFEV_1_) indicated better lung function, lower sweat chloride concentrations and a higher Cystic Fibrosis Questionnaire-Revised respiratory domain (CFQ-R RD) score indicating less respiratory symptoms and a better overall quality of life (Zaher et al., 2021). Furthermore, an observational study in PwCF homozygous for the F508del mutation ranging from 20.8 to 48.3 years old (median age = 31.1 years) taking TRIKAFTA^®^ over the course of 48 weeks was done (Carnovale et al., 2022). Although inflammation was not evaluated, an improvement in FEV_1_, body mass index and sweat chloride was observed. On the other hand, a decrease in exacerbation and a need for intravenous antibiotics was reported. However, it is important to note that due to COVID-19, CF clinics have seen less bacterial exacerbations since patients were isolating and wearing masks. This is relevant given it is a retrospective study (i.e., without adequate control group comparisons) (Carnovale et al., 2022).

While the disease results in the occurrence of many symptoms, lung deterioration caused by repeated bacterial, viral infections, and exacerbations are the main cause of mortality and morbidity (Ratjen et al., 2015). To treat these infections, a combination of anti-bacterial, antifungal, and antiviral medications is needed, but cause complications after repeated and extended periods of use (Hahn et al., 2018). Therefore, novel treatment options that can mitigate the genetic defects of the CFTR protein while also addressing the inflammatory storm seen in PwCF, still need to be explored. Interestingly, a study by Gentzsch et al. found that the rescue of mutant CFTR was improved upon CFTR modulator therapies *in vitro* undergoing airway epithelial inflammation (Gentzsch et al., 2021). This could prove to be very intriguing and would be interesting to see in an *in vivo* model.

An interesting strategy, which has gained much ground across various fields in the last decade, is the repurposing and combination of drugs, in the hope of achieving additive and even synergistic effects in treating diseases. In CF disease, small molecule combination has been the strategy employed by the pharmaceutical industries and academic laboratories, which yielded the most successful results (Lopes-Pacheco, 2019; Villella et al., 2019; Pinto et al., 2021).

It was therefore important to investigate whether the inflammation-controlling (pro-resolution) treatment, LAU-7b, would be complementary to the recently approved TRIKAFTA^®^ therapy and not interfere with the efficacy of the combination in mice with CF lung disease. With the eventual aim of improving the quality of life for PwCF and to address a broader range of the pathology observed in CF lung disease, we have assessed the efficacy of the combination of pro-resolution LAU-7b, with modulator and corrector therapies.

This study examined the impacts that combinatory treatment of LAU-7b (oral fenretinide) and a Triple therapy similar to TRIKAFTA^®^ have *in vivo* on DD mice, and also *in vitro,* using a CFBE overexpressing F508del cell line. While each treatment on its own has demonstrated ameliorative effects in the studies conducted thus far, their combination is studied here for the first time. TRIKAFTA^®^’s mechanism of action remains to be fully elucidated. However, it is thought that correctors, Elexacaftor and Tezacaftor, directly interact with the mutant CFTR protein, facilitating its movement and trafficking to the Golgi, while the potentiator Ivacaftor, increases channel activity at the membrane level (Zaher et al., 2021). LAU-7b′s mechanism of action was previously discussed; functioning as a membrane lipid modulator and exerting inflammation-controlling (pro-resolution) in multiple *in vitro* and *in vivo* systems (Garić et al., 2017; Garić et al., 2020b). Given our understanding of the two treatments and the benefits they each have, we began by studying their combination *in vivo* in our older, 14 to 20-month-old DD and WT mice. Before studying the effect of various infections on the efficacy of TRIKAFTA^®^, it was important to first study mice that develop CF lung disease even under pathogen-free conditions. This occurs when CF mice reach an age corresponding to that of PwCF at which those with a class 1 and 2 mutations in the CFTR gene have a decline in the force expiratory volume in 1 s (FEV_1_).

After diagnosis of CF lung disease, a standard measure of disease progression and airway obstruction is FEV_1_ tested by spirometry (Szczesniak et al., 2017). Declining pulmonary function among PwCF is age-dependent, however, the most dramatic drop in FEV_1_ occurs between puberty and the age of 30, although it is dependent on the CFTR gene mutation. The decline in lung function over time in CF patients results from chronic airway inflammation and mucus dehydration, which are triggered by infection-induced increases in pro-inflammatory lipid mediators, which are regulated by AA release (Miele et al., 1997; Elizu et al., 2008). In mice, an invasive measure of airway resistance is a gold standard assay performed to monitor pulmonary function. In Figure 1A, our WT control group showed an expected slight increase upon MCh addition. However, a significant impairment in airway function is seen for the DD NT and VEH treated mice, most notably at the 100 mg/ml MCh dose, upon comparison to WT control (Figure 1B). Moreover, a significant improvement is seen upon treatment with LAU-7b, which shows airway resistance levels comparable to healthy WT controls. While Triple therapy treatment improves airway function, its effects are less marked than those seen in LAU-7b treated animals. The mice treated with the combination of Triple therapy and LAU-7b, displayed a significant improvement in lung function. The normalization of lung function upon combinatory treatment denotes a lower airway hyperresponsiveness, like that of WT control mice. This indicates that mice treated with LAU-7b, or LAU-7b + Triple therapy, have significantly better pulmonary function than mice treated with only Triple therapy.

In PwCF, the abnormal mucosal defences facilitate recurrent chronic infection, most often with *P. aeruginosa* and sometimes with *Aspergillus fumigatus*, which negatively impacts the inflammatory milieu leading to lung damage and eventually lung failure, the main cause of morbidity and mortality in PwCF (Leclair and Hogan, 2010; Ratjen et al., 2015; Cottrill et al., 2020). It is well documented that MUC5AC overexpression plays a key role in airway plugging, and both MUC5AC and MUC5B are not only upregulated in chronically infected PwCF but are further augmented during lung exacerbations that happen in a large percentage of PwCF 3–4 times a year (Rowe et al., 2005; Fahy and Dickey, 2010; Garić et al., 2020a). To improve mucus clearance, mucoactive agents, consisting of mucolytics and hyperosmolar agents, can be used (Hurt and Bilton, 2014). Bronchodilators work to improve airway opening by relaxing the smooth muscles in the bronchial wall facilitating mucus clearance (Smith and Edwards, 2017; Smith et al., 2020). Despite various treatments, delayed resolution of inflammation that frequently occurs in PwCF following exacerbation, often results in permanent lung damage. To assess lung deterioration in our F508del mice that were kept in a specific pathogen free condition (SPF), histological analysis of lung sections stained with H&E was done (Figure 3). Histology revealed overall ameliorated pathological parameters in mice treated with LAU-7b and LAU-7b in combination with Triple therapy as compared to vehicle treated controls, further supporting the results obtained for the physiological assessment. Hyperplasia of airways with significantly thickened airway lining is a pathological feature of cystic fibrosis and other respiratory diseases (Gorrieri et al., 2016). In Figure 3, a major difference can be seen between the airways of the WT control mouse and the DD-NT or DD-VEH mouse. Both DD-NT and DD-VEH mice have elevated levels of hyperplasia compared to WT control. These results corroborate the findings of a lower airway resistance in WT mice, and higher resistance in DD NT and VEH treated mice, highlighting the drastic difference in overall pulmonary health for these two groups. While treatment with LAU-7b or Triple therapy reduce airway cell hyperplasia, the combination of LAU-7b and Triple therapy yields superior results compared to Triple therapy alone, with uniform looking lungs, comparable to those of WT controls. The histology images are further supported by Figure 3G, displaying the lowest number of infiltrating cells for the combinatory treatment group.

In response to allergic reactions or infections, goblet cells present in the airways start production of mucus, and an increase in the reproduction rate of these cells leads to mucus hypersecretion, leading to abnormal mucus accumulation and airway plugging. This results in decreased antimicrobial functions and impaired mucociliary clearance, which can further worsen the CF condition (Puchelle et al., 2002). Histological assessment of lung sections demonstrates that treatment with LAU-7b and Triple therapy dramatically diminish the thickening of the airway lining. As the inflammatory response in the lungs of PwCF is self-perpetuating and can be worsened by abnormal immune activity, it is important that any developing CF treatment considers the likely possibility of goblet cell hyperplasia and its consequences. Mucin expression that is inducible upon allergic response to Aspergillus antigens and infections with *P. aeruginosa*, *Staphylcoccus aureus* and other bacterial and fungal induced lung pathology was not evaluated in the current study, but is one of the important aspects of our ongoing studies.

Since oxidative markers, fatty acids, and overall lipid profiles, are known to be dysregulated in both CF mice and PwCF from birth, prior to their development of CF lung disease (Strandvik et al., 2001; Strandvik, 2010; Youssef et al., 2020), further analysis was done using a portion of the liver, lung and plasma of WT and DD mice under the various treatments to obtain the lipidomic profile of those mice, first, oxidative stress markers were analyzed. Being a universal biological response, oxidative stress plays a major role in a variety of inflammatory disease conditions. In CF, neutrophils are continuously recruited to the airways and liberate their toxic products such as oxidants, in an uncontrolled fashion (Hector et al., 2014). While antioxidants shield the lung from free oxidative damage in healthy patients, the amount and duration of neutrophilic inflammation overwhelms these defence systems in CF subjects, leading to increased protein and lipid oxidation in the lungs. To improve the defense system in PwCF, it is important to introduce a treatment which reduces levels of lipid and protein oxidation as marked by MD and 3-NT, respectively. In Figure 4, LAU-7b treatment in DD mice significantly reduced levels of MD and 3-NT in the lungs, plasma, and liver, to a greater extent than Triple therapy treatment. However, upon combination with Triple therapy, improvements are comparable to that of LAU-7b alone, lowering the levels of those oxidative markers below the WT threshold. Thus, the combination of Triple therapy with LAU-7b, is significantly better than Triple therapy alone, given LAU-7b′s antioxidant properties counteracting the overwhelming recruitment of neutrophils seen in lung inflammation.

Moreover, fatty acids abnormalities, in AA, DHA, and EPA, are consistently reported in CF (Sinaasappel et al., 2002). These abnormalities create an imbalance in the DHA/AA ratio in favour of AA, contributing to the increase in pulmonary inflammation and mucus dehydration, resulting in deterioration of PwCF’s condition. However, the causal connection between the expression of CFTR protein and this phenomenon has rarely been examined. Some evidence that suggests a role for CFTR in fatty acid metabolism as it was shown in cell culture models, where CFTR dysfunction results in defective fatty acid composition (Andersson et al., 2008). Furthermore, similar polyunsaturated fatty acid changes in CF affected organs such as the lung, pancreas and ileum have been reported in CFTR knock-out mice, further suggesting a causal link between CFTR and fatty acid metabolism (Freedman et al., 1999). Further studies also report that modulation of saturated fatty acids correlates with the modulation of LCCs and VLCCs (Garić et al., 2020a). In Figure 5, EPA and DHA levels are shown in the lungs, plasma and liver of DD mice. Upon treatment with LAU-7b, a significant increase in those omega-3 fatty acids was observed. While treatment with Triple therapy alone did show some improvement, it is only after its combination with LAU-7b that we see an increase in EPA and DHA comparable and even exceeding levels of EPA and DHA seen in WT mice. Combinatory treatment with Triple therapy and LAU-7b shows normalization of AA levels, which is not seen with Triple therapy treatment alone. Figures 6 and 7 show LCCs and VLCCs levels in lung, liver and plasma, respectively. In 2020, Liessi et al. performed an untargeted lipidomic analysis on CFBE41o-cells upon various treatment groups, which included the Triple combination therapy (VX-661/VX-445/VX-770) (Liessi et al., 2020). Our results, as seen in Figures 6 and 7 corroborate their findings (Figure 4), of a downregulation in C14:0 and C16:0 and a concomitant upregulation in VLCCs (Liessi et al., 2020). Recently, an interesting study was published by Westholter and others reporting ceramide levels obtained from plasma analysis of 25 PwCF (age 35.56 ± 12.75; 20 out of 25 with intermittent or chronic Pseudomonas infection) treated for 4 weeks with TRIKAFTA® (Westhölter et al., 2022). A very modest improvement (decrease) in C16:0 ceramide levels (0.218 ± 0.09 before treatment and 0.178 ± 0.06 (*p* = 0.0051) after 4 weeks of treatment) with TRIKAFTA® was reached. Furthermore, a very modest improvement in the levels of C24:0 (1.354 ± 0.47 before and 1.674 ± 0.65 after 4 weeks of treatment (*p* = 0.0048)) was also achieved. However, only 3 PwCF out of 25 reached levels for C24:0 of 2.5 after treatment, with none of the patients reaching a level of 4, which is typical for healthy individuals (Garić et al., 2017). No improvement in C22:0 or C24:1 ceramide was reported following the treatment with TRIKAFTA® and no analysis of C26:0 ceramides was done. The ratio between C16Cer/C24Cer has improved from 0.171 ± 0.06 before treatment to 0.112 ± 0.03 after 4 weeks of TRIKAFTA® treatment (*p*-value = <0.001) (Westhölter et al., 2022).

In Figure 8 the VLCC/LCC ratio, which is known to be altered in CF disease, is emphasized. While Triple therapy can partially improve this ratio by increasing VLCC levels and decreasing LCC levels, the greatest correction occurs upon combinatory treatment with LAU-7b, surpassing the WT threshold in lungs, liver, and plasma of WT mice. Overall, Triple therapy alone seems to be beneficial in terms of correcting the pool of fatty acids, and ceramides, however, the combination of Triple and LAU-7b is demonstrated to be superior in further ameliorating these levels to similarity with what is seen in WT mice. This cooperative effect might be particularly important for PwCF since most of them are chronically infected with bacteria such as *P. aeruginosa*, and/or fungi.

Results obtained *in vitro* corroborate the *in vivo* findings, once again demonstrating that the combination of Triple with LAU-7b is more efficacious in improving the VLCC/LCC ratio of the CFBE41o-(F508del) cell line, than Triple therapy alone. Interestingly, our data demonstrated that fenretinide treatment increases total CFTR protein levels (Figure 9), in the same cell line, thereby providing more protein that can be subsequently processed to the cell membrane with the help of modulators and the potentiator, enabling functional recovery of CFTR channel.

Ultimately, there is a benefit for PwCF from TRIKAFTA®. Moving forward, it is imperative to study whether the effect of TRIKAFTA® is long-lasting and whether the lung inflammation resulting from recurrent lung infections does not diminish its efficacy over time, as previously seen with Kayldeco®, a similarly effective CFTR modulator (Heltshe et al., 2018). The combination of Triple therapy and LAU-7b might resolve this important facet. Taken together, the results obtained in this study strongly suggest for a potential clinical benefit from using TRIKAFTA® in combination with LAU-7b, with the aim of slowing the lung degradation and further improving overall quality of life in PwCF.

While the study presented above can have a lasting impact for PwCF, there are limitations. The cost of the Triple treatment was a limiting factor in our decision for the number of mice to include in each treatment group. Therefore, we chose to include 3 mice in each Triple and Triple + LAU-7b groups, given it is the minimum number of mice required to achieve statistical significance. Furthermore, kinetics of inflammatory mediators and mucin induction, which is usually following exposure to lung pathogens or their filtrates, could not be evaluated in this study since animals were maintained in a specific pathogen-free (SPF) condition. The airway thickening quantification in Figure 3G is based on a semi-quantitative assessment of four airways per mouse lung at ×20 magnification and establishment of a fully quantitative approach would be desirable in the future.

## Data Availability

The original contributions presented in the study are included in the article/Supplementary Material, further inquiries can be directed to the corresponding author.

## Dedication

This manuscript is dedicated to the memory of our dear friend and colleague Dr. Bob Scholte.
