# Supplementary material for: Treatment With LAU-7b Complements CFTR Modulator Therapy by Improving Lung Physiology and Normalizing Lipid Imbalance Associated With CF Lung Disease
Source: Front Pharmacol. 2022 May 20;13:876842. doi: 10.3389/fphar.2022.876842 (PMC9163687; doi:10.3389/fphar.2022.876842)
Supplement: Supplementary file 1 [file DataSheet1.PDF]

Supplementary Material

1 Supplementary Figures

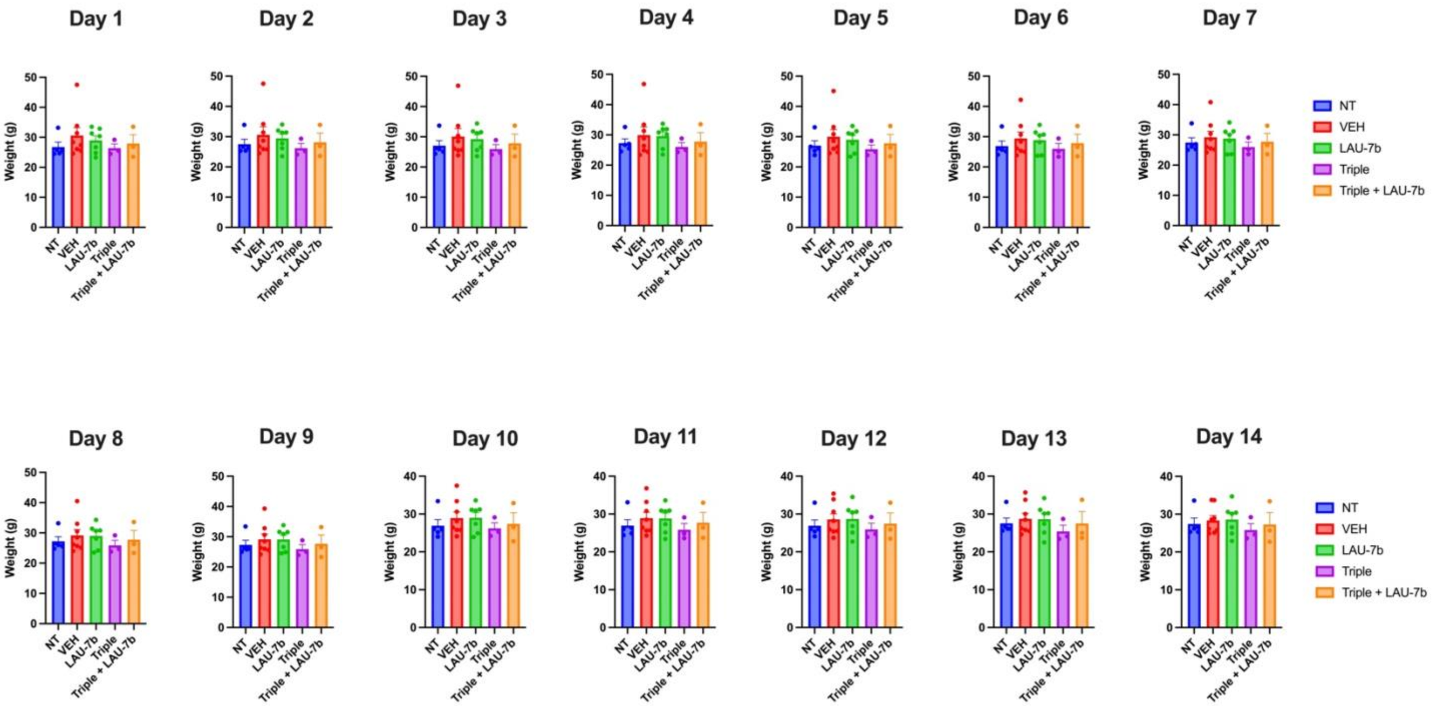

Supplementary Figure 1. F508del/F508del (DD) mice weights during 14-day treatment. Mice were weighed every day during their 14-day treatment. No statistical significance was seen among treatment groups. n=3-8 mice per group.

DD NT

<u>8–10-month-old mice</u>

Male

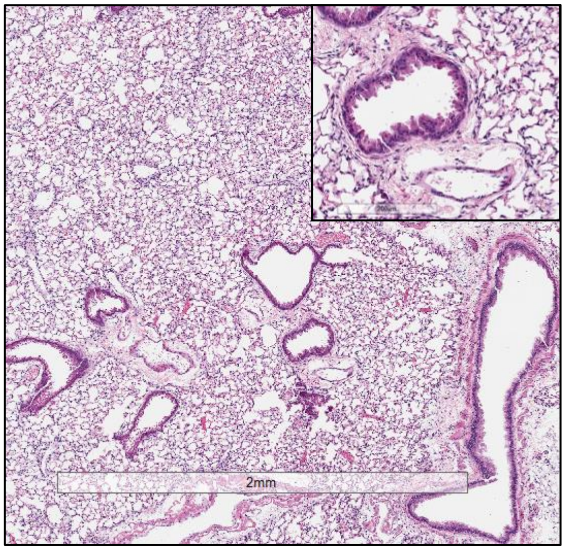

Female

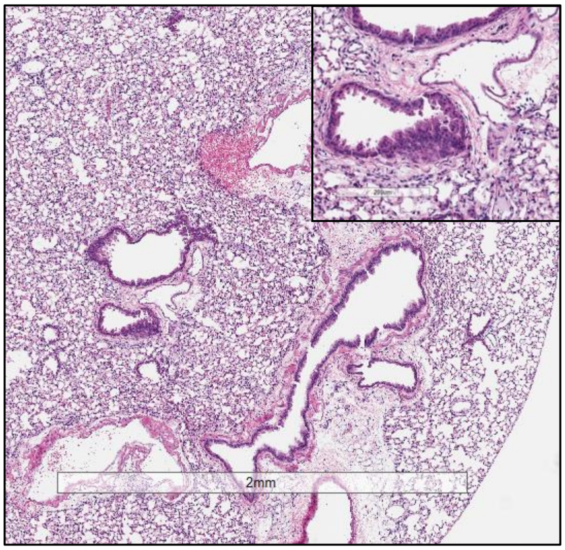

<u>14-20-month-old mice</u>

Male

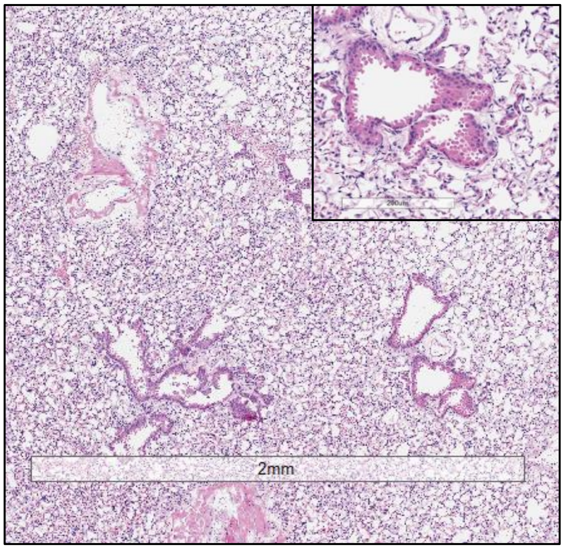

Female

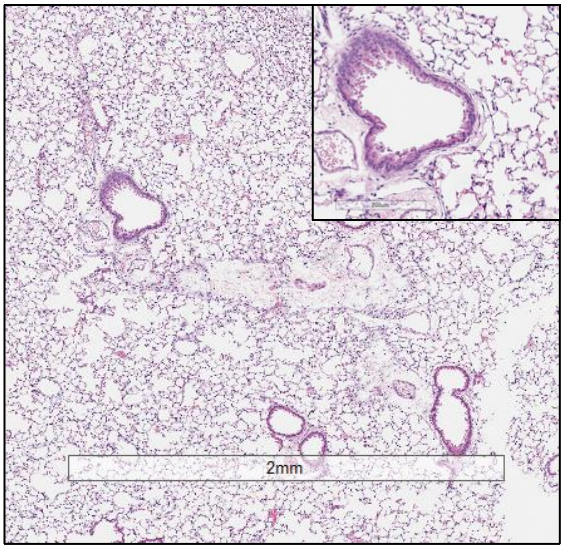

WT NT

<u>9-month-old mouse</u>

Male

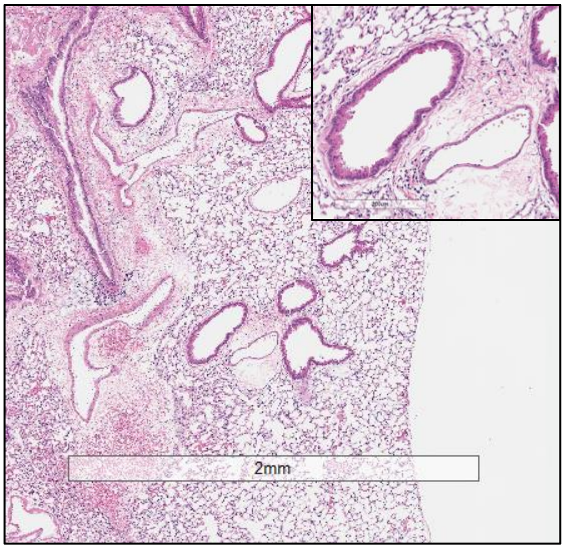

<u>14-month-old mouse</u>

Male

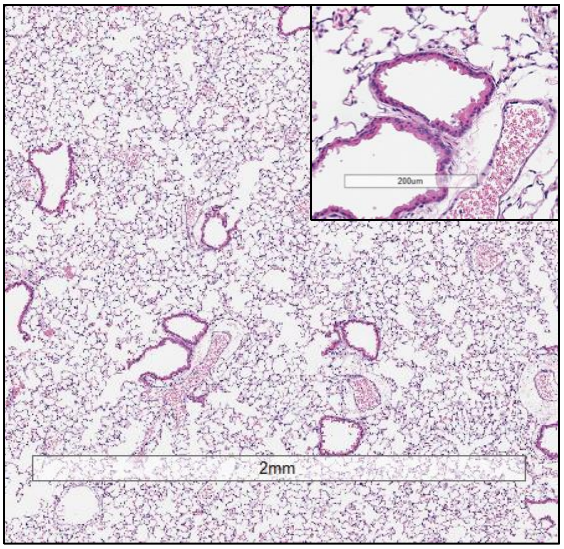

Supplementary Figure 2. Airway hyperplasia in males and females of different ages seen in DD and WT NT mice.

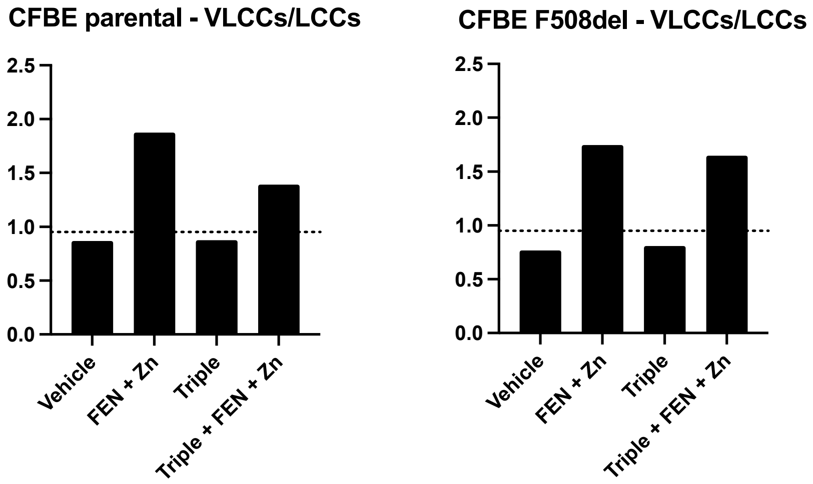

Supplementary Figure 3. Combination of Triple therapy, LAU-7b and Zinc improves VLCCs/LCCs ratios in CFBE41o-(P) and (F508del) cell lines compared to CFBE41o-(WT) baseline (dotted line).

Supplementary Table 1. Table of One-Way ANOVA analysis of airway resistance comparing saline (S) and 100 mg/ml MCh (M).

Dunnett's T3 multiple comparisons test	Mean Diff.	95.00% CI of diff.	Summary	Adjusted P Value
S - WT control vs. M - WT control	-1.472	-2.195 to -0.7493	***	0.0001
S - WT control vs. M - DD VEH & NT	-4.213	-7.734 to -0.6923	*	0.0193
S - WT control vs. M - DD LAU-7b	-1.392	-2.324 to -0.4597	**	0.0043
S - WT control vs. M - DD Triple + LAU-7b	-1.540	-2.841 to -0.2389	*	0.0166
S - DD VEH & NT vs. M - WT control	-1.367	-2.223 to -0.5111	***	0.0006
S - DD VEH & NT vs. M - DD VEH & NT	-4.108	-7.535 to -0.6808	*	0.0176
S - DD VEH & NT vs. M - DD LAU-7b	-1.287	-2.280 to -0.2940	**	0.0066
S - DD VEH & NT vs. M - DD Triple + LAU-7b	-1.435	-2.780 to -0.08981	*	0.0316
S - DD LAU-7b vs. M - WT control	-1.633	-2.615 to -0.6509	***	0.0004
S - DD LAU-7b vs. M - DD VEH & NT	-4.374	-7.843 to -0.9049	*	0.0129
S - DD LAU-7b vs. M - DD LAU-7b	-1.553	-2.639 to -0.4666	**	0.0024
S - DD LAU-7b vs. M - DD Triple + LAU-7b	-1.701	-3.092 to -0.3104	**	0.0097
S - DD Triple + LAU-7b vs. M - WT control	-1.945	-3.442 to -0.4475	*	0.0165
S - DD Triple + LAU-7b vs. M - DD VEH & NT	-4.686	-8.194 to -1.178	**	0.0089
S - DD Triple + LAU-7b vs. M - DD LAU-7b	-1.865	-3.354 to -0.3756	*	0.0169
S - DD Triple + LAU-7b vs. M - DD Triple + LAU-7b	-2.013	-3.647 to -0.3790	*	0.0136
